# Rapid-onset massive immune-mediated pericardial effusion in a patient with anti-IFN-γ autoantibodies syndrome

**DOI:** 10.3389/fimmu.2026.1894750

**Published:** 2026-07-22

**Authors:** Fushou Chen, Yan Ning, Hanlin Liang, Jiaqi Qin, Siqiao Liang, Xuemei Huang, Xiaona Liang, Feixiang Tan, Ni Chen, Siyao Wu, Limei Hong, Zhiyi He, Tao Feng

**Affiliations:** 1 Department of Respiratory and Critical Care Medicine, Wuming Hospital of Guangxi Medical University, Nanning, Guangxi, China; 2 Department of Respiratory and Critical Care Medicine, The First Affiliated Hospital of Guangxi Medical University, Nanning, Guangxi, China

**Keywords:** anti-IFN-γ autoantibodies syndrome, case report, immune dysregulation, nontuberculous mycobacteria, pericardial effusion, Talaromyces marneffei

## Abstract

**Background:**

Anti-interferon-gamma (IFN-γ) autoantibodies (AIGAs) syndrome is an emerging adult-onset immunodeficiency (AOID) associated with opportunistic infections and immune dysregulation. Pericardial effusion as a direct manifestation of this syndrome is rarely reported.

**Case presentation:**

A 46-year-old man with a history of *Talaromyces marneffei* (TM) infection and clinically suspected pulmonary *nontuberculous mycobacterial* (NTM) disease presented with rapid-onset massive pericardial effusion. The NTM diagnosis was not microbiologically confirmed, as the patient declined bronchoscopy. Pericardial fluid analysis revealed a high inflammatory cell count and an anti-IFN-γ autoantibody titer of 1:2500, identical to serum, despite a negative microbiological workup and no definitive evidence of malignancy on available cytological evaluation. The patient responded well to corticosteroids and anti-NTM therapy, with complete resolution of effusion.

**Conclusion:**

This case highlights pericardial effusion as a potential immune-mediated complication of AIGAs syndrome. Clinicians should consider this diagnosis in patients with unexplained serositis and a history of opportunistic infections.

## Introduction

AIGAs syndrome is a newly recognized AOID, predominantly reported in Southeast Asia (1). It is characterized by impaired cellular immunity, leading to recurrent or severe opportunistic infections, particularly with intracellular pathogens such as *Mycobacterium avium complex* and *Talaromyces marneffei* (1–3). Immune-mediated organ damage may also occur, although pericardial involvement is rare.

Pericardial effusion is a common clinical finding with a broad differential diagnosis, including infection, malignancy, autoimmune disease, and idiopathic causes (4, 5). In developing countries, tuberculosis remains the leading cause, whereas in developed countries, viral and idiopathic etiologies predominate (4). We report a case of rapid-onset massive pericardial effusion in a patient with AIGAs syndrome, in whom no infectious cause was identified and no malignancy was detected on the available evaluation, and who responded dramatically to corticosteroid therapy.

## Case presentation

**Figure 1:**
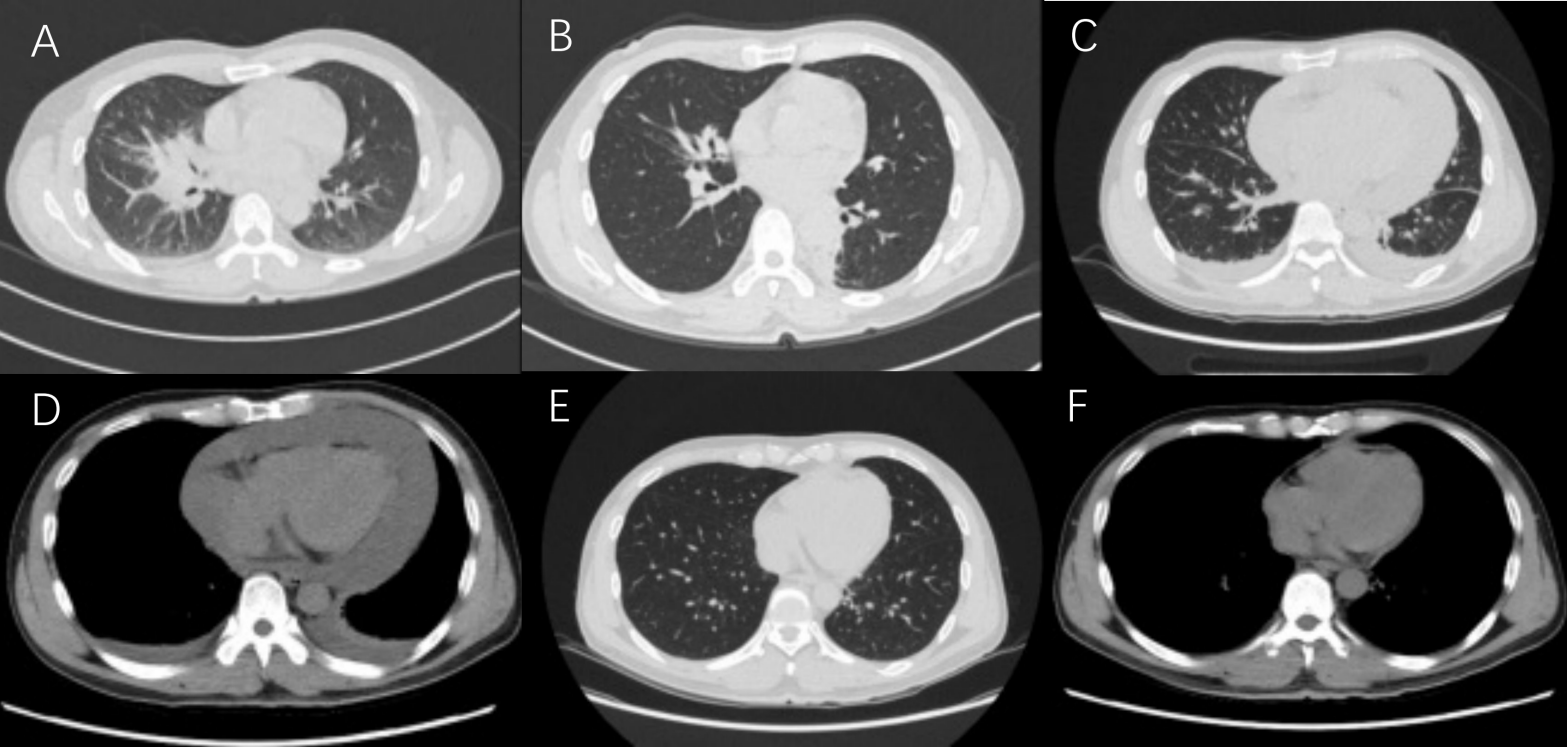
Chest CT findings. **(A)** Initial CT (September 2023) demonstrates patchy opacities in the right lung, multiple enlarged right hilar and mediastinal lymph nodes, and small bilateral pleural effusions. **(B)** Follow-up CT (January 2024) reveals significant resolution of the pulmonary opacities. **(C)** Subsequent CT (March 2025, lung window) indicates progression of consolidations in both lower lobes. **(D)** Mediastinal window at the same level shows a notable increase in pleural and pericardial effusions. **(E)** The latest CT after treatment shows marked regression of lung infiltrates. **(F)** Mediastinal window demonstrates near-complete resolution of pleural and pericardial effusions.

A 46-year-old man was admitted to the First Affiliated Hospital of Guangxi Medical University on September 11, 2023, presenting with an 11-day history of fever, cough, and productive sputum. Initial chest CT on admission (September 2023) demonstrated patchy opacities in the right lung, multiple enlarged right hilar and mediastinal lymph nodes, and small bilateral pleural effusions, raising strong clinical suspicion of opportunistic infection. To establish a definitive etiological diagnosis, bronchoscopy with bronchoalveolar lavage fluid (BALF) collection was performed. Acid-fast bacilli smear and bacterial and fungal cultures of BALF were all negative, and no malignant cells were detected. Metagenomic next-generation sequencing (mNGS) of BALF identified *Talaromyces marneffei* (sequence count: 40934) and human herpesvirus 5 (CMV) (sequence count: 19558), leading to a diagnosis of *Talaromycosis* with involvement of the lungs, multiple systemic lymph nodes, and bones. AIGAs were detected in peripheral blood by enzyme-linked immunosorbent assay (ELISA) at a titer of 1:2500. Antifungal therapy with voriconazole (200 mg twice daily) was initiated, which yielded some clinical improvement; however, a persistent cough prompted further investigation. Given the clinical presentation and the known susceptibility of AIGAs patients to multiple intracellular pathogens, a concurrent pulmonary NTM infection was clinically suspected, and an empirical anti−NTM regimen was initiated, although microbiological confirmation was not available because the patient declined bronchoscopy. This regimen was selected based on local NTM epidemiology, clinical guideline recommendations, and the need to avoid strong drug-drug interactions between rifampin and voriconazole. The addition of a combination anti-NTM regimen—azithromycin, linezolid, and moxifloxacin—resulted in significant symptomatic relief. The patient was subsequently maintained on long-term combination therapy with voriconazole, azithromycin, moxifloxacin, and linezolid. A follow-up chest CT scan on January 6, 2024, showed partial resolution and reduction of the pulmonary infectious lesions compared to prior imaging (**Figures 1A, B**). After discussion with the care team regarding the risks of early treatment discontinuation, the patient independently decided to discontinue all medications in February 2024 (**Table 1**).The re-examination showed a decreased titer of AIGAs at 1:500. The patient has no history of structural heart disease.

**Table 1:** Timeline of clinical course, AIGAs titers, treatments, and outcomes.

Time point	Clinical events	AIGAs titer	Anti-infectious treatments	Corticosteroid regimen	Outcome
Sep–Oct 2023	Initial admission (Sep 2023) with fever, cough, and productive sputum; CT: right hilar mass, lymphadenopathy, small pleural effusions; BALF mNGS: TM + CMV. Discharged in Oct 2023 on voriconazole + empirical anti-NTM regimen.	Serum: 1: 2500	Voriconazole; Empirical anti-NTM (linezolid, moxifloxacin, azithromycin)	—	Fever resolved; clinical improvement; discharged on maintenance therapy
Jan 2024	Follow-up CT: pulmonary lesions significantly reduced	Serum: 1: 500	Continued same regimen	—	Sustained improvement
Feb 2024	Treatment completion; all medications discontinued	—	—	—	—
Feb 2025	Readmission with cough, shortness of breath, and fever; CT: progression of lower lobe lesions, new right pleural effusion	Serum: 1: 2500	Empirical anti-NTM (linezolid, moxifloxacin, azithromycin)	—	Symptoms improved; discharged
Mar 2025	Presented with chest pain, dyspnea, and back pain; echocardiography: moderate-to-large pericardial effusion; pericardiocentesis (Mar 18): exudative fluid, high inflammatory cells, negative for infection/malignancy	Serum: 1: 2500; Pericardial: 1: 2500	Linezolid + rifampin + ethambutol	Prednisone 30 mg/d initiated during hospitalization	Rapid clinical and radiological improvement; near-complete resolution of effusion
Apr 2025	Follow-up CT: significant resolution of pericardial and pleural effusions; lung lesions decreased	—	Continued anti-NTM + tapered prednisone	Apr 8: tapered to 20 mg/d	Near-complete resolution; discharged on maintenance therapy
May–Jun 2025	May 13: chest pain, pale red rash on right chest/back (resolved after rifampin discontinuation); rifampin discontinued due to drug rash; azithromycin and voriconazole added.	1:500 (Jun 10, 2025)	Linezolid + ethambutol + azithromycin + voriconazole + prednisone (rifampin discontinued on May 13)	May 13: tapered to 15 mg/d; Jun 10: maintained at 15 mg/d	Rash gradually resolved
Jun–Aug 2025	No cough, dyspnea, or chest pain; clinically stable	1:500 (Aug 10, 2025)	Linezolid + ethambutol + azithromycin + voriconazole	Jun 25: tapered to 10 mg/d; maintained until Aug 12, 2025	Stable; no recurrence of pericardial effusion
After Aug 2025	Lost to follow-up	—	—	—	No further follow-up data available

AIGAs, anti-IFN-γ autoantibodies; BALF, bronchoalveolar lavage fluid; CMV, cytomegalovirus; CT, computed tomography; mNGS, metagenomic next-generation sequencing; NTM, nontuberculous mycobacteria; TM, *Talaromyces marneffei*. Dashes indicate that corticosteroid therapy was not administered or AIGAs titer was not measured at that time point. The patient was lost to follow-up after August 12, 2025.

Physical examination on admission revealed normal vital signs, and the patient was afebrile. No enlarged superficial lymph nodes were palpable. A small amount of moist rales was heard in both lung fields. Upon admission, laboratory tests revealed leukocytosis (WBC 16.63×10^9^/L) with elevated absolute neutrophil count (NEUT# 11.69×10^9^/L) and absolute eosinophil count (EOS# 1.28×10^9^/L). Liver function tests showed hypoalbuminemia (ALB 35.5 g/L) and hyperglobulinemia (GLB 45.0 g/L). Markers of systemic inflammation were elevated, including erythrocyte sedimentation rate (ESR 75 mm/h), C-reactive protein (CRP 77.4 mg/L), and procalcitonin (PCT 0.276 ng/mL). Immunoglobulin G (IgG) was elevated (34.02 g/L), and AIGAs were detected in peripheral blood by ELISA at a titer of 1:2500. Cardiac enzymes and renal function parameters were within normal limits. A non-contrast chest CT scan demonstrated slight progression of lower lobe pulmonary lesions, mild mediastinal lymphadenopathy, and a new small right pleural effusion compared to the prior study from January 16, 2024. Ultrasonography of the right neck identified multiple enlarged lymph nodes. Bronchoscopy was deferred due to patient refusal.

**Figure 2:**
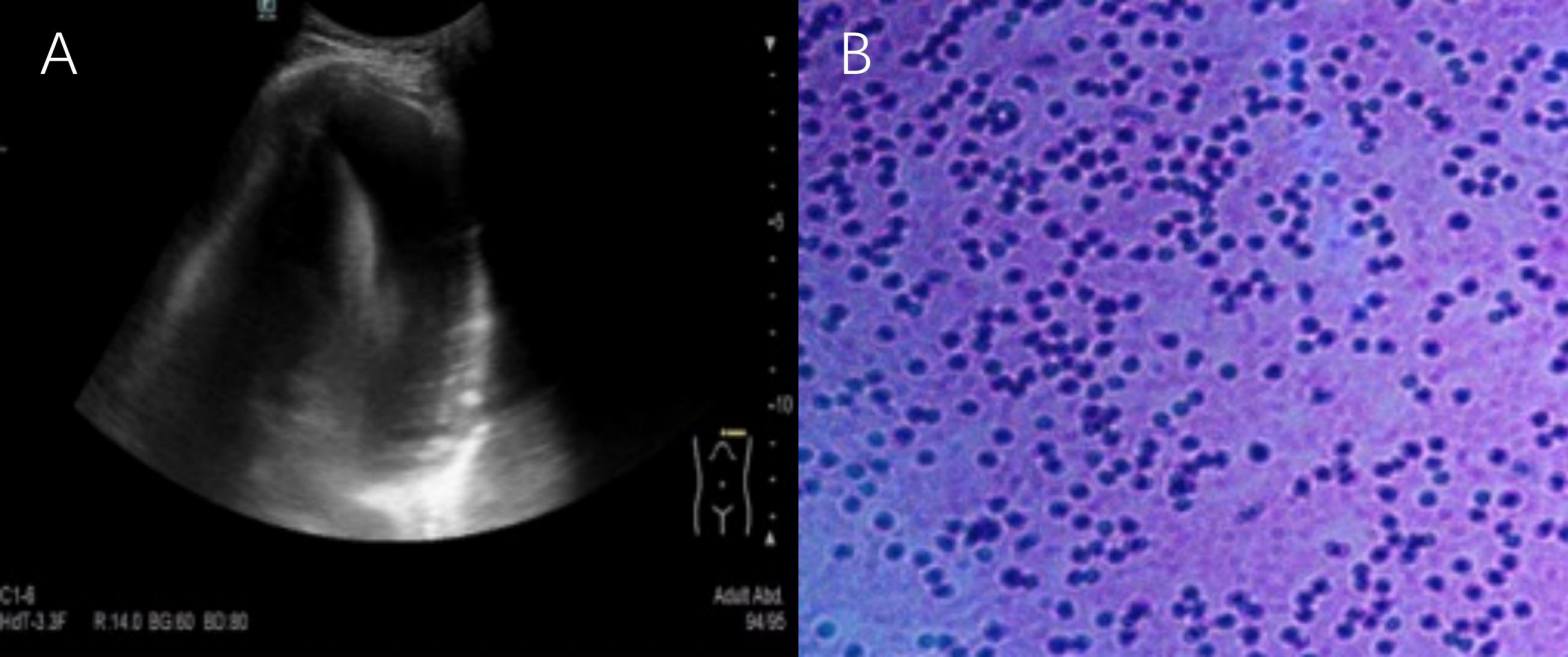
Characteristics of pericardial effusion. **(A)** Ultrasound shows a large amount of pericardial effusion. **(B)** Cytological analysis of the pericardial fluid reveals predominantly inflammatory cells with no evidence of malignancy.

**Figure 3:**
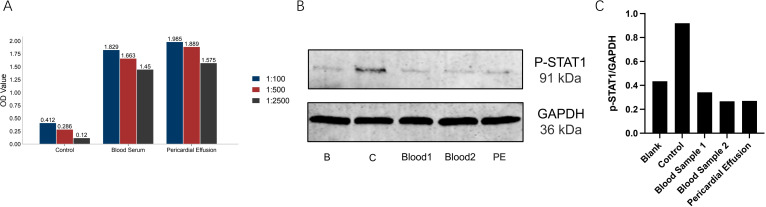
Detection of anti-IFN-γ autoantibodies (AIGAs) and P-STAT1 expression in serum and pericardial effusion. **(A)** ELISA analysis of AIGAs optical density (OD) values. Both serum and pericardial effusion samples showed high-titer positivity at a 1:2500 dilution, while blank and negative controls remained negative. **(B)** Western blot analysis of P-STAT1 protein expression, with GAPDH as a loading control. P-STAT1 bands were detected in both pericardial effusion and serum samples at a 1:2500 dilution; no specific bands were observed in blank or negative control samples. **(C)** Quantitative analysis of P-STAT1 protein expression normalized to GAPDH. Compared to controls, P-STAT1 expression was significantly reduced in both serum and pericardial effusion samples, supporting the neutralizing activity of AIGAs.

Given the clinical and radiological progression suggestive of NTM reactivation, and the patient’s repeated refusal of bronchoscopy precluding microbiological confirmation, an empirical anti-NTM regimen consisting of linezolid, moxifloxacin, and azithromycin was re−initiated. During this treatment, he reported chest tightness; an electrocardiogram revealed QT interval prolongation. Azithromycin and moxifloxacin were subsequently discontinued, and therapy was adjusted to linezolid, rifampin, and ethambutol, supplemented with prednisone acetate 30 mg daily for its anti-inflammatory effect. His clinical status improved, and he was discharged. On March 11, 2025, the patient represented with chest pain exacerbated by expiration, accompanied by back pain and dyspnea. Although initially responsive to analgesics, the symptoms recurred and progressively intensified. Evaluation at a local hospital, including chest CT and echocardiography, revealed lower lobe pulmonary inflammation and a moderate pericardial effusion, prompting referral to our center (**Figures 1C, D**). At our institution, echocardiography confirmed a massive pericardial effusion (**Figure 2A**). Blood tests indicated persistent inflammation, with leukocytosis (12.11×10^9^/L, 62.8% neutrophils), elevated CRP (78.31 mg/L), an ESR of 82 mm/h, an IgG level of 24.73 g/L, and an IgG4 level of 8.30 g/L. Electrocardiography was normal. Following assessment of contraindications, ultrasound-guided pericardiocentesis with catheter drainage was performed on March 18, 2025. The aspirated fluid was brown-red and turbid. Analysis confirmed an exudative profile with a markedly elevated nucleated cell count (4.62 × 10^9^/L), predominantly mononuclear cells (68%). Biochemical analysis showed elevated total protein (66.80 g/L) and lactate dehydrogenase (LDH 224 U/L), but a normal adenosine deaminase (ADA) level (9 U/L). Cytological examination of the pericardial fluid revealed abundant inflammatory cells with no evidence of malignancy on the available cytological evaluation (**Figure 2B**). Several tumor markers within the fluid were significantly elevated: CA-125 (922.40 U/mL), ferritin (2, 434.05 ng/mL), and CYFRA 21-1 (63.27 ng/mL). Routine microbiological studies, including acid-fast bacilli smear and bacterial culture, were negative. NGS of the fluid detected *Torquetenovirus* (TTV) sequences (24 reads). The AIGAs titer in the pericardial fluid was 1:2500, consistent with that in serum (**Figure 3A**). Western blot (WB) analysis further confirmed the presence of AIGAs in both samples. P-STAT1 bands were detected in both pericardial effusion and serum samples at a 1:2500 dilution, with GAPDH as a loading control; no specific bands were observed in blank or negative control samples (**Figure 3B**). Quantitative analysis revealed that P-STAT1 expression was significantly reduced in both serum and pericardial effusion samples compared to controls (**Figure 3C**), supporting the neutralizing activity of AIGAs and the consistency of titers between the two compartments.

Given the clinical context of a suspected nontuberculous mycobacterial infection and a profoundly dysregulated immune response characterized by high-titer AIGAs, the pericardial effusion was attributed to an immune-mediated process. Management was reinstated with combination antimicrobial therapy (linezolid, rifampin, and ethambutol) alongside immunosuppression with prednisone acetate 30 mg daily (**Table 1**). The patient’s condition improved satisfactorily, leading to discharge on a regimen of linezolid, rifampin, ethambutol, and prednisone acetate (**Figures 1E, F**).

### Laboratory assays

Sample collection and processing Peripheral venous blood was obtained from the patient after overnight fasting on the first day of admission. Pericardial effusion was collected during the initial ultrasound-guided pericardiocentesis before any intra-pericardial intervention. All specimens were centrifuged within 1 hour after collection, aliquoted, and stored at −80 °C without freeze-thaw cycles prior to analysis. Pooled serum from age- and sex-matched healthy controls was processed and stored under identical conditions.

AIGAs detection and neutralization assay Serum and pericardial fluid AIGAs titers were measured by ELISA. A cell-based functional neutralization assay was performed to verify the biological activity of AIGAs. Briefly, recombinant human IFN-γ (100 U/mL) was preincubated with an equal volume of diluted (1:2500) patient serum, pericardial effusion supernatant, or healthy control serum for 1 hour at 37 °C. The mixture was then used to stimulate THP-1 monocytic cells. Intracellular phosphorylated STAT1 (Tyr701) expression was detected by Western blotting, with GAPDH as the loading control. Reduced P-STAT1 expression indicated neutralizing activity of AIGAs.

## Discussion

This report presents a case of rapidly progressive, massive, immune-mediated pericardial effusion complicating AIGAs syndrome, thereby expanding the recognized clinical spectrum of this disorder. AIGAs syndrome is an adult-onset immunodeficiency characterized by recurrent severe opportunistic infections with intracellular pathogens like TM and NTM, stemming from high-titer neutralizing autoantibodies that impair Th1 immunity (1). Beyond infection susceptibility, autoantibody-associated immune-mediated organ damage, affecting systems such as the skin and eyes, is increasingly acknowledged (6–8). Notably, our case was unique for its presentation as acute serositis primarily involving the pericardium during a clinically stable phase, without evidence of active infection or malignancy.

The etiology of pericardial effusion is broad. After excluding infectious, neoplastic, cardiac, and common autoimmune causes, idiopathic pericarditis is often diagnosed (5, 9, 10). In our patient with established AIGAs syndrome, determining the underlying etiology of pericardial effusion was particularly challenging. Nonetheless, several independent lines of evidence collectively support an immune-mediated pathogenesis over an infectious origin. First, the normal pericardial ADA level effectively excludes tuberculous pericarditis—a leading cause of exudative effusions in tuberculosis−endemic regions. Second, the effusion profile was exudative, with elevated protein and LDH, and a mononuclear predominance, all of which are characteristic of sterile serositis rather than bacterial infection. Third, the presence of equally high titers of AIGAs in both peripheral blood and pericardial fluid points to a localized immune process, likely driven by *in situ* production or sequestration of autoreactive antibodies. Fourth, comprehensive microbiological investigation, including culture and mNGS, failed to identify any known pathogen; the sole detected sequence, TTV, is a commensal virus commonly regarded as a biomarker of immune dysfunction rather than a causative agent of serositis. Finally, the most compelling evidence derives from the therapeutic response: the patient’s symptoms and radiographic abnormalities resolved dramatically within days of corticosteroid initiation—a temporal trajectory that strongly favors the resolution of an immune-mediated inflammatory process over the clearance of an untreated infection. We acknowledge, however, that the absence of positive microbiological findings does not entirely exclude an infectious process, particularly for fastidious or intracellular organisms, and especially given the patient’s prior exposure to corticosteroids.

Key differential diagnoses, particularly IgG4-related disease (IgG4-RD), were meticulously evaluated. Although serum IgG4 was elevated, the absence of characteristic histopathology (e.g., storiform fibrosis, obliterative phlebitis, dense IgG4+ plasma cell infiltration) or other organ involvement ruled out IgG4-RD (11, 12). Other systemic autoimmune diseases were also excluded.

This case underscores that in patients with AIGAs syndrome, immune-mediated serositis should be considered in the differential diagnosis of acute serous effusions unexplained by active infection or malignancy. While the precise mechanisms warrant further study, our experience demonstrates that adding immunomodulatory therapy (e.g., glucocorticoids) to control underlying infections can yield rapid clinical improvement, potentially avoiding unnecessary invasive procedures or excessive antimicrobial therapy.

We acknowledge several limitations in this case. First, genetic testing for inherited immunodeficiencies was not performed due to financial constraints, and we cannot completely exclude the possibility of an underlying genetic predisposition that may have contributed to the patient’s susceptibility to opportunistic infections. The patient was counselled on the clinical value of genetic testing for future follow-up. Second, the diagnosis of pulmonary NTM disease was based on clinical suspicion rather than microbiological confirmation, as the patient declined bronchoscopy; consequently, mycobacterial cultures, species identification, and PCR assays were not available, and the NTM species could not be determined. Third, the antimicrobial regimen (azithromycin, linezolid, and moxifloxacin) was selected empirically based on local treatment practices for NTM in AIGAs patients and the susceptibility profile of NTM in our region, rather than on individualized drug susceptibility testing. Fourth, the patient had received oral prednisone acetate prior to diagnostic pericardiocentesis, which may have confounded the interpretation of the pericardial fluid analysis, as systemic corticosteroids can suppress local inflammation and immune responses, potentially altering the cellular composition and biochemical profile of the effusion. Fifth, comprehensive screening for other cytokine autoantibodies was not performed; however, the patient’s characteristic clinical phenotype of disseminated TM and NTM infections, together with high−titer AIGAs and functional neutralizing activity, is highly specific for AIGAs syndrome. Future studies should incorporate broader cytokine autoantibody profiling. Sixth, this is a single−case report, and the findings may not be generalizable to other patients. Finally, while we attributed the pericardial effusion to an immune−mediated process, we acknowledge that negative cultures and mNGS cannot completely exclude an infectious etiology, particularly in the setting of prior corticosteroid use and the inherent limitations of culture−based and molecular diagnostics for fastidious or intracellular organisms. Despite these limitations, this case report provides several strengths: it expands the recognized clinical spectrum of AIGAs syndrome, demonstrates comprehensive pericardial fluid evaluation, and offers practical guidance for clinicians managing similar cases.

## Conclusion

Pericardial effusion represents an under-recognized, potentially immune-mediated complication of AIGAs syndrome. In patients presenting with unexplained serositis—particularly those with a history of opportunistic infections—evaluation for AIGAs is warranted. Early diagnosis and initiation of immunomodulatory therapy can improve clinical outcomes and may avert unnecessary invasive diagnostic procedures.

## Data Availability

The datasets presented in this study can be found in online repositories. The names of the repository/repositories and accession number(s) can be found in the article/supplementary material.
